# Optimized assessment for establishing myocardial viability prior to revascularization of a chronic total coronary occlusion using cardiac magnetic resonance imaging

**DOI:** 10.1186/1532-429X-11-S1-P195

**Published:** 2009-01-28

**Authors:** Sharon WM Kirschbaum, Alexia Rossi, Eric Boersma, Dirk J Duncker, Martin van den Ent, Gabriel P Krestin, Patrick W Serruys, Pim J de Feyter, Robert-Jan M van Geuns

**Affiliations:** Erasmus MC, Rotterdam, Netherlands

**Keywords:** Percutaneous Coronary Intervention, Cardiac Magnetic Resonance, Dobutamine, Single Parameter, Myocardial Viability

## Introduction

Predictive value of delayed enhancement CMR is moderate especially in segments with an intermediate transmural extent of infarction (TEI).

## Purpose

We sought to improve the predictive value of cardiac magnetic resonance imaging (CMR) using combined viability assessment in patients before percutaneous coronary intervention (PCI) of a chronic total coronary occlusion (CTO).

## Methods

We studied patients with a successful (43/71) and without a successful (29/71) PCI of a CTO. Segmental wall thickening (SWT) was quantified before and after PCI. Before PCI, using CMR, 5 viability indexes were evaluated: TEI, contractile reserve during dobutamine, end diastolic wall thickness (EDWT), unenhanced rim thickness and SWT of the unenhanced rim (SWTur). We determined predictive value for improvement in SWT>10% after PCI for each viability index and for combined viability assessment.

## Results

Mean SWT improved significantly in patients with successful PCI (16 ± 19% to 39 ± 35 %; p < 0.0001), mean SWT did not improve in patients without successful PCI (19 ± 21% to 21 ± 25%; p = 0.54). Predictive value for each viability index is presented in Table 1. Combined viability assessment using contractile reserve during dobutamine, SWTur and TEI, improved the predictive value with a sensitivity of 92%(95% CI 82–97) and specificity of 83%(95% CI 70–91).

**Table 1:** Diagnostic performance of each viability index for the prediction of improvement in SWT.

	Sensitivity (%)	Specificity (%)	PPV (%)	NPV (%)	Accuracy (%)
LDD (>7%)	94 (88–98)	77 (63–87)	89 (82–94)	87 (73–95)	89(83–94)
TEI (<50%)	89 (81–94)	50(36–64)	79 (70–85)	68 (51–82)	76(69–83)
EDWT (>6 mm)	92 (84–96)	27 (16–41)	72 (64–79)	61 (39–80)	71(63–78)
Unenhanced rim thickness (>3 mm)	94(88–98)	48 (34–62)	79 (71–85)	81 (62–92)	79(73–86)
SWTUR (<45%)	88 (80–93)	33 (21–47)	73 (64–80)	57(38–74)	69(62–76)

LDD, contractile reserve during low dose dobutamine; TEI, transmural extent of infarction; EDWT, end diastolic wall thickness; SWTur, Segmental wall thickening of the unenhanced rim; PPV, positive predictive value; NPV, negative predictive value; 95% confidence interval is presented between brackets.

## Conclusion

Combined viability assessment was superior to viability assessment using a single parameter. This may be useful for the selection of patients for PCI of a CTO.

